# Effects of feeding corn containing an alpha-amylase gene on the performance and digestibility of growing cattle

**DOI:** 10.1093/tas/txac013

**Published:** 2022-01-28

**Authors:** Marissa Ann Glaser, Sean P Montgomery, Chris I Vahl, Evan C Titgemeyer, Callie S Kubick, Grant I Glaser, Tyler J Spore, William R Hollenbeck, Ross A Wahl, Dale A Blasi

**Affiliations:** 1 Department of Animal Sciences and Industry, Kansas State University, Manhattan, KS 66506, USA; 2 Corn Belt Livestock Services, Papillion, NE 68046, USA; 3 Department of Statistics, Kansas State University, Manhattan, KS 66506, USA

**Keywords:** Enogen Feed Corn, alpha-amylase, growing cattle, corn silage, dry-rolled corn

## Abstract

Two growth performance studies and two digestibility trials were conducted to evaluate the effects of feeding Enogen Feed Corn silage and corn grain to growing cattle. In Exp. 1, there were a total of four diets offered for ad libitum intake. The four diets consisted of two varieties of corn (Enogen Feed Corn [EFC] vs. yellow #2 corn [CON]) with two different methods of corn processing (dry-rolled [DR] vs. whole-shelled [WS]) and were formulated to provide 1.13 Mcal NEg/kg dry matter (DM); corn grain was 28.6% of diet DM. Average daily gain (ADG) and ending body weight tended to be greater for calves fed EFC than for those fed CON (*P* < 0.10). Gain:feed (G:F) was better for calves fed EFC (*P* < 0.01), improving by 5.5% over calves fed CON. In Exp. 2, a digestibility trial was conducted using seven cannulated Holstein steers fed the same diets from Exp. 1. Ruminal pH was not affected by corn variety (*P* > 0.82). Liquid passage rate was greater for CON-fed calves and associated with lower digestibility. Total tract DM and organic matter (OM) digestibilities were greater for EFC-fed calves (*P* < 0.04). In Exp. 3, there were four diets offered for ad libitum intake. Dietary factors were arranged as a 2 × 2 factorial and consisted of two hybrids of corn silage (EFC silage [EFC-S] vs. control silage [CON-S]) and two varieties of corn grain (EFC grain [EFC-G] vs. control [CON-G]; both were dry-rolled). Diets were formulated to provide 1.11 Mcal NEg/kg DM; corn grain was 38.5% of diet DM, and corn silage was 40% of diet DM. ADG was 6.0% greater (*P* < 0.01) and G:F was numerically (*P* < 0.14) 3.3% greater for calves fed EFC-S than for those fed CON-S, but substituting EFC-G for CON-G did not affect ADG or G:F. In Exp. 4, a digestibility trial was conducted using eight cannulated beef steers fed the same diets as Exp. 3. Liquid passage rate (*P* > 0.20), ruminal pH (*P* > 0.23), and ruminal total volatile fatty acid concentrations (*P* > 0.27) were unaffected by treatment. Total tract digestibilities of DM and OM were numerically greater by 2.5% and 2.2%, respectively, for calves fed the EFC-S compared with those fed CON-S. Feeding a corn hybrid containing alpha-amylase enzyme improved G:F of growing calves. Feeding EFC can benefit the beef industry by allowing less processing of grain without sacrificing performance.

## Introduction

Although the amylolytic activity of ruminal microbes is able to increase twofold with the addition of grains to the diet, this increase is relatively minor in comparison to the use of exogenous amylases (Rojo et al., 2005). Therefore, the use of external amylase enzymes has the potential to increase the efficiency of starch digestion more than manipulation of the activity of microbes in the rumen (Rojo et al., 2005). Data on feeding a corn hybrid containing an alpha-amylase enzyme to cattle is limited, and previous research involving supplementing exogenous alpha-amylase in cattle diets has been variable (Tricarico et al., 2007; Tricarico et al., 2005; Hristov et al., 2008; DeFrain et al., 2005). However, Jolly-Breithaupt et al. (2019) in two separate experiments showed that feeding Enogen Feed Corn (EFC) to feedlot cattle improved gain:feed (G:F) by 5.5% and 5.7%. The relative value of EFC as a source of energy either as a silage and/or grain for newly arrived and growing beef cattle is unknown.

The most important issue surrounding the use of corn hybrids containing amylase is the digestibility and energy value of the grain. However, the presence of the amylase enzyme might also allow less processing of grains to be utilized with the grain still maintaining greater digestion and energy concentration. Also, amylase provided to the diet through the corn might not only improve the digestibility of the amylase-containing corn directly, but the amylase also might improve digestion of starch from other feed ingredients.

The objectives of these experiments were to 1) compare effects of feeding EFC on growing cattle performance; 2) compare effects of feeding EFC on digestibility and ruminal parameters of growing cattle; 3) compare effects of feeding EFC silage and EFC grain on growing cattle performance; and 4) compare effects of feeding EFC silage and EFC grain on digestibility and ruminal parameters of growing cattle.

## Materials and Methods

All procedures involving the use of animals were approved by the Kansas State University Institutional Animal Care and Use Committee.

### Experiment 1. Performance Study

A total of 426 English crossbred steers (body weight = 244 kg ± 90 kg) were purchased from Oklahoma, Texas, and Missouri and assembled at a farm in Lazbuddie, Texas and then shipped 909 km to the Kansas State University Beef Stocker Unit on May 15, 2017. The steers were used in a completely randomized design with a 2 × 2 factorial arrangement of treatments to examine the effects of feeding two corn types (Enogen Feed Corn [EFC] vs. yellow #2 corn [CON]) with two methods of corn processing (dry-rolled [DR] vs. whole-shelled [WS]) on the performance of stocker cattle in a 91-d receiving and growing study. Single sources of EFC and CON were used to produce the respective DR and WS products. Particle size was analyzed by the Kansas State University Swine Lab (Manhattan, KS) for DR for both corn types and averaged 1,633 and 1,920 microns for EFC and CON, respectively. The four treatment diets (EFC/DR, EFC/WS, CON/DR, and CON/WS) were formulated to provide 1.13 Mcal NEg/kg of DM as well as to meet requirements for minerals, vitamins, and ruminally available protein (Table 1). Diets contained (DM basis) 28.6% corn, 6.4% supplement, 17.5% alfalfa hay, 17.5% prairie hay, and 30% wet distillers grains (Golden Triangle Energy Cooperative, Craig, MO). Wet distillers grains was utilized as a protein and energy source (Corrigan et al., 2009). All diets were offered for ad libitum intakes. The EFC containing the alpha-amylase enzyme was provided by Syngenta Seeds, LLC (Downers Grove, IL). All diets had similar starch content.

**Table 1.** Ingredient and nutrient composition of diets (Exp. 1 and 2)^1^

Ingredient, % of DM	Corn grain source^2^			
	CON		EFC	
	Corn processing^3^			
	DR	WS	DR	WS
Yellow #2 corn grain, dry rolled	28.57	—	—	—
Yellow #2 corn grain, whole shelled	—	28.57	—	—
Enogen Feed Corn grain, dry rolled	—	—	28.57	—
Enogen Feed Corn grain, whole shelled	—	—		28.57
Wet distillers grains	30.00	30.00	30.00	30.00
Alfalfa hay	17.50	17.50	17.50	17.50
Prairie hay	17.50	17.50	17.50	17.50
Supplement^4^	6.43	6.43	6.43	6.43
Nutrient composition, % of DM				
Exp. 1				
Dry matter, %	57.9	57.6	54.3	53.2
Crude protein	17.4	17.3	18.2	18.7
Neutral detergent fiber	27.4	29.1	30.1	30.6
Acid detergent fiber	17.3	19.1	17.5	19.1
Starch	25.5	26.6	23.0	25.8
Calcium	1.16	1.18	1.08	1.19
Phosphorus	0.42	0.45	0.42	0.47
Exp. 2				
Dry matter, %	59.9	59.1	56.7	58.0
Crude protein	17.3	17.9	18.4	18.5
Neutral detergent fiber	29.0	28.1	30.0	29.4
Acid detergent fiber	16.6	15.7	16.8	17.2
Starch	25.5	26.6	23.0	25.8
Calcium	1.16	1.17	1.14	1.15
Phosphorus	0.42	0.43	0.45	0.44

Diets were formulated to contain 1.74 Mcal NEm/kg DM and 1.13 Mcal NEg/kg DM (NASEM, 2016).

CON, control; EFC, Enogen Feed Corn.

DR, dry rolled; WS, whole shelled.

Supplement pellet was formulated to contain (DM basis) 11.09% crude protein, 8.50% calcium, 0.42% phosphorus, 5.50% salt, 0.80% potassium, 0.57% magnesium, 1.70% fat, 11.04% acid detergent fiber, and 331 mg/kg lasalocid (Bovatec; Zoetis, Parsippany, NJ).

Upon arrival, calves were individually weighed using a hydraulic squeeze chute on load cells (Silencer, Moly Manufacturing Inc., Lorraine, KS) and given an individual visual identification ear tag and a radio-frequency identification (RFID) button tag. Steers were then divided over 32 pens and provided long-stem hay and ad libitum access to water via automatic waterers until the next morning. Thirty-two steers on the lower end of the weight spectrum and 10 steers on the higher end of the weight spectrum were removed from the research population. Following processing on the day after arrival (day 0), the remaining 384 steers were stratified by individual arrival weight and sorted to 32 pens each containing 12 steers. Pens were then assigned randomly to one of four treatments, which equaled eight pens per treatment. Pens were soil surfaced and of equal size (9.1 × 15.2 m) with concrete bunks measuring 9.1 m in length attached to a 3.6-m apron.

The morning after arrival (day 0), calves were weighed individually, ear tagged with a pen number, and vaccinated for viral and clostridial diseases. The day-0 body weights were used as the initial body weights for the experiment. Vision 7 Somnus with Spur (Merck Animal Health, Omaha, NE) was used for protection against clostridial pathogens. Pyramid 5 + Presponse (Boehringer Ingelheim Vetmedica Inc., St. Joseph, MO), a modified-live vaccine protecting against infectious bovine rhinotracheitis (IBR), bovine viral diarrhea types 1 and 2 (BVDI-II), parainfluenza 3 (PI3), and bovine respiratory syncytial virus (BRSV), was used for protection against respiratory pathogens. The calves were also treated for internal parasites with Safe-Guard containing 10% fenbendazole (Merck Animal Health, Madison, NJ). On day 21, all research animals were revaccinated for respiratory diseases with Bovishield Gold 5, a modified-live virus vaccine protecting against IBR, BVDI-II, BRSV, and PI3 (Zoetis, Parsippany, NJ).

Animals were fed their respective diets once daily at approximately 0700 h using a Roto-Mix feed wagon (model 414-14B), which was thoroughly cleaned between each diet. Feed delivery was adjusted based on daily refusals to ensure ad libitum intakes without excess of unconsumed feed. Individual animal weights were measured on day −1 (arrival), day 0 (initial processing), day 21 (revaccination), day 56/57 (fecal grab sampling), and day 91 (ending weights). Fecal samples were obtained individually from steers in 16 pens on day 56 and individually from steers in the remaining 16 pens on day 57, pooled by pen, and analyzed for starch (Richards et al., 1995) and DM (by drying at 105 °C) the same week by a commercial laboratory (SDK Laboratories; Hutchinson, KS). Combined weights of all cattle from a single pen were measured on days 7, 14, 35, 63, and 77. Individual ingredient samples were collected weekly and composited for analysis, and total mixed ration samples from each treatment were collected weekly and analyzed individually (dry matter [DM], crude protein [N × 6.25; AOAC International, 1997], acid detergent fiber (ADF; Van Soest et al., 1991), neutral detergent fiber (NDF; Van Soest, 1991), starch (Richards et al., 1995), calcium [Bowers and Rains, 1988], and phosphorus [AOAC International, 1997]; Table 1) by SDK Laboratories.

Animals were observed each day for signs of morbidity, such as depression, decreased appetite, and nasal or ocular discharge. Steers showing any of these signs were removed from the pen and herded to the treatment area. Once restrained in the chute, rectal temperature was measured and a clinical illness score (CIS) was assigned. Clinical illness score was assessed on a scale of 1 to 4: 1 = normal and healthy; 2 = slightly ill with mild depression/gauntness; 3 = moderately ill with severe depression/labored breathing/ocular or nasal discharge; and 4 = severely ill to the point of death with little response to human approach. Animals with a rectal temperature > 39.9 °C and a CIS > 1 were treated. Treatment protocol was as follows: first treatment, Resflor Gold (300 mg/mL florfenicol and 16.5 mg/mL flunixin meglumine; Merck Animal Health, Madison, NJ); second treatment, Baytril 100 (100 mg/mL enrofloxacin; Bayer Animal Health, Shawnee Mission, KS); third treatment, Biomycin (200 mg/mL oxytetracycline; Boehringer Ingelheim Vetmedica, Inc., St. Joseph, MO). At the third treatment, animals were considered chronic and removed from the research population.

### Experiment 2. Intake and Digestibility Study

Seven ruminally cannulated Holstein steers (body weight = 198 ± 10 kg) were used in an incomplete 4 × 4 Latin rectangle design to determine diet digestibility and digestion characteristics. Data from one steer in the second period was removed due to issues with the rumen cannula. Experimental diets were the same as in Exp. 1 (Table 1). The study consisted of 4 consecutive 15-d periods consisting of a 10-d diet adaptation, 4-d fecal collection, and 1 d for ruminal fluid sampling. As the loads of feed were mixed daily for Exp. 1, the amount needed for Exp. 2 was removed from the beginning of each load; feed samples were analyzed independently from those in Exp. 1 (Table 1).

Animals were housed in individual outdoor pens (12.2 × 15.2 m). Each steer had ad libitum access to tank waterers, which were filled daily. Animals were fed once daily at approximately 1000 h. Diets were fed for ad libitum intake to target at least a 10% refusal. Total mixed ration samples were collected on days 10 through 14 and composited for each period for analysis. Total mixed ration and weekly individual ingredient samples from Exp. 1 overlapping with the sampling week were sent to SDK Laboratories for nutrient analysis (DM, ash [Undersander, 1993], crude protein, ADF, NDF, starch, calcium, and phosphorus). On days 4 through 14, chromium oxide (Cr_2_O_3_; 10 g/d) was top-dressed and hand mixed into each animal’s diet as a marker to calculate digestibility (Titgemeyer, 1997). Refusals were collected on days 11 through 15 and composited for each animal for each period. Fecal samples were collected on days 11 through 14 from the rectum of the steers every 8 h with the sampling time moved forward by 2 h each day so that every 2-h interval after feeding was represented. Fecal samples were frozen (−20 °C) and stored for later analysis. Refusal and fecal samples were composited for each steer in each period and sent to SDK Laboratories for analysis (DM, ash, crude protein, ADF, NDF, starch, calcium, and phosphorus).

Refusal samples were dried at 55 °C, air equilibrated, and ground through a 1-mm screen using a Wiley mill. Fecal samples were dried at 105 °C and ground through a 1-mm screen using a Wiley mill. Fecal and refusal samples were weighed (0.5 g) into 50-mL crucibles and ashed in a muffle oven at 600 °C for 4 h. Chromium concentrations were determined by atomic absorption spectrophotometry (Williams et al., 1962).

On day 15 of each period, ruminal fluid samples were collected from four different locations in the rumen at 0, 2, 4, 6, 8, 12, 18, and 24 h after feeding and pooled within sampling time. Following the 0-h sampling, 3 g of cobalt-EDTA (Co-EDTA; 0.4 g Co; Udén et al., 1980) dissolved in 200 mL of water was dosed into the rumen. Rumen samples were analyzed for pH with a pH meter (Orion Model 230A; Beverly, MA) and strained through eight layers of cheesecloth. Strained rumen fluid was pipetted into four 2-mL micro-centrifuge tubes containing 0.25 mL of *m*-phosphoric acid and then frozen at −20 °C for later analysis of volatile fatty acid (VFA) concentrations by gas–liquid chromatography and ammonia (Broderick and Kang, 1980). Additionally, 20 mL of strained rumen fluid was collected and frozen at −20 °C for later analysis of Co concentration to determine liquid passage rate. Cobalt concentrations were analyzed by atomic absorption spectrophotometry. Liquid passage rate (Merchen, 1988) was determined by regressing the natural logarithm of ruminal Co concentrations at 2 through 18 h after Co-EDTA dosing against time for each steer in each period using the nonlinear procedure in SAS (SAS Inst. Inc., Cary, NC). Passage rate was determined as the negative slope of the line.

### Experiment 3. Performance Study

A total of 362 crossbred steers of Tennessee origin (body weight = 298 ± 75 kg), previously backgrounded for 63 d on a common diet at the Kansas State University Beef Stocker Unit, were used in a completely randomized 2 × 2 factorial design to determine the effects of feeding 2 hybrids of corn silage (EFC, hybrid E111F1-5122A-EZT0 [EFC-S] vs. Mycogen corn, hybrid TMF14L46 [CON-S]) and two varieties of corn grain (EFC [EFC-G] vs. yellow #2 corn grain [CON-G]) on the performance of stocker cattle in a 91-d growing study. Both corn grain types, EFC-G and CON-G, were dry-rolled before feeding. Particle size was analyzed by the Kansas State University Swine Lab for both corn grains and averaged 2,628 microns for EFC-G and 3,206 microns for CON-G. The four treatment diets (CON-G/CON-S, EFC-G/CON-S, CON-G/EFC-S, and EFC-G/EFC-S) were formulated to contain 1.11 Mcal NE_g_/kg DM and contained 38.5% corn, 7.5% supplement, 7.5% alfalfa hay, 7.5% prairie hay, and 40% corn silage (Table 2). All diets were offered for ad libitum intake for 77 d. This was followed by a gut-fill equalization period of 14 d at the end of the trial (days 77 to 91), when all animals were limit-fed at 2.2% of body weight daily a common diet (Table 3).

**Table 2.** Ingredient and nutrient composition of diets (Exp. 3 and 4)^1^

Ingredient, % of DM	Corn silage hybrid^2^			
	CON-S		EFC-S	
	Corn grain source^3^			
	CON-G	EFC-G	CON-G	EFC-G
Mycogen corn silage^4^	40.0	40.0		
Enogen Feed Corn silage^5^	—	—	40.0	40.0
Yellow #2 corn grain, dry rolled	38.5	—	38.5	-
Enogen Feed Corn grain, dry rolled	-	38.5	—	38.5
Alfalfa hay	7.0	7.0	7.0	7.0
Prairie hay	7.0	7.0	7.0	7.0
Supplement^6^	7.5	7.5	7.5	7.5
Nutrient composition, % of DM				
Exp. 1				
Dry matter, %	50.9	51.3	54.6	54.3
Crude protein	13.4	13.4	13.0	13.2
Neutral detergent fiber	27.9	27.9	25.8	28.1
Acid detergent fiber	19.0	18.7	17.2	18.0
Starch	35.8	36.2	39.3	37.1
Calcium	0.88	0.90	0.81	0.86
Phosphorus	0.31	0.31	0.28	0.28
Exp. 2				
Dry matter, %	52.9	51.2	54.7	54.7
Crude protein	12.9	12.9	12.9	12.8
Neutral detergent fiber	28.1	29.1	27.8	27.6
Acid detergent fiber	19.0	19.2	18.2	18.1
Starch	39.1	38.5	39.1	39.3
Calcium	0.80	0.79	0.80	0.79
Phosphorus	0.29	0.29	0.28	0.28

Diets were formulated to contain 1.72 Mcal NEm/kg DM and 1.11 Mcal NEg/kg DM (NASEM, 2016).

CON-S, control corn silage; EFC-S, Enogen Feed Corn silage.

CON-G, control corn grain; EFC-G, Enogen Feed Corn grain.

Contained 30.0% dry matter and 28.7% starch.

Contained 34.4% dry matter and 34.7% starch.

Supplement pellet was formulated to contain (DM basis) 8.80% crude protein, 5.68% calcium, 1.00% phosphorus, 3.78% salt, 1.89% potassium, 0.47% magnesium, 3.08% fat, 11.9% acid detergent fiber, and 231 mg/kg monensin (Rumensin; Elanco, Greenfield, IN).

**Table 3.** Ingredient and nutrient composition of gut-fill equalization diet (Exp. 3)

Ingredient	% of DM
Yellow #2 corn, dry rolled	38.82
Sweet Bran (Cargill Animal Nutrition, Blair, NE)	40.00
Alfalfa hay	6.50
Prairie hay	6.50
Supplement^1^	8.18
Composition, % of dry matter	
Dry matter, %	70.8
Crude protein	16.2
Neutral detergent fiber	25.0
Acid detergent fiber	12.0

Supplement pellet was formulated to contain (DM basis) 8.80% crude protein, 5.68% calcium, 1.00% phosphorus, 3.78% salt, 1.89% potassium, 0.47% magnesium, 3.08% fat, 11.9% acid detergent fiber, and 231 mg/kg monensin (Rumensin; Elanco, Greenfield, IN).

Twenty-five acres of dryland EFC silage (Enogen Feed Corn, E111F1-5122A-EZT0) and 6.5 acres of dryland CON silage (Mycogen, TMF14L46) were harvested in August of 2017 at 2/3 milk-line, chopped to a length of 20 mm, kernel processed to 2 mm with an on-board processor, and bagged (SILOBOLSA Plastar Premium silage bags; Buenos Aires, Argentina) the same day using a 550 horsepower Versa bagger (Astoria, OR). At harvest, EFC and CON silage averaged 34% and 29% DM, respectively. Each silage hybrid was ensiled for approximately 147 d. CON silage and EFC silage yielded approximately 24.7 and 20.2 Mg/hectare, respectively.

The 10 heaviest steers were removed from the research population. The remaining 352 steers were stratified by weight and assigned randomly to pens containing 11 animals. The 32 pens were then randomly allocated to one of four treatments, with eight pens per treatment. Pens were the same as described in Exp. 1. On day −6, calves were allocated to pens based on individual body weight measured using a hydraulic squeeze chute with load cells (Silencer, Moly Manufacturing Inc., Lorraine, KS). On day 0, calves were individually weighed and tagged with a pen number. All calves were vaccinated for viral and clostridial diseases at the start of the previous 63-d backgrounding phase at the Kansas State University Beef Stocker Unit. Vision 7 Somnus with Spur (Merck Animal Health, Omaha, NE) was used for protection against clostridial pathogens, and Bovishield Gold 5 (Zoetis, Parsippany, NJ), a modified-live vaccine protecting against infectious bovine rhinotracheitis (IBR), bovine viral diarrhea types 1 and 2 (BVDI-II), parainfluenza 3 (PI3), and bovine respiratory syncytial virus (BRSV), was used for protection against respiratory pathogens. Zuprevo 18% (180 mg/mL tildipirosin; Merck Animal Health, Madison, NJ) was used as a metaphylaxis on arrival for the treatment of bovine respiratory disease (BRD) associated with *Mannheimia haemolytica*, *Pasteurella multocida*, and *Histophilus somni*. The calves were also treated for internal parasites with Safe-Guard containing 10% fenbendazole (Merck Animal Health, Madison, NJ) at the start of the previous 63-d backgrounding phase.

The steers were fed their respective diets once daily at approximately 0700 h using a Roto-Mix feed wagon (model 414-14B). Feed delivery was adjusted based on daily refusals to ensure ad libitum intakes without an excess of left-over feed. Individual ingredient samples were collected weekly and composited for analysis and total mixed ration samples from each diet were collected weekly and analyzed individually (DM, crude protein, ADF, NDF, starch, calcium, and phosphorus; Table 2) by SDK Laboratories. Individual animal weights were measured on day -6 (allocation), day 0 (initial processing), day 49 (fecal grab sampling), and day 91 (ending weights). Fecal samples were obtained individually on day 49 and sent to SDK Laboratories for analysis of starch and DM the same week. Combined weights of all cattle from a single pen were measured on days 14, 28, 42, 56, 70, 77, and 91. Animals were observed daily for morbidity and treated according to the protocol from Exp. 1.

### Experiment 4. Intake and Digestibility Study

Eight ruminally cannulated, predominantly Angus, beef steers (body weight = 211 ± 30 kg) were used in a 4 × 4 Latin rectangle design to determine diet digestibility and digestion characteristics. Data from one steer were removed from the first period due to rumen cannula issues. Experimental diets were the same as in Exp. 3 (Table 2). The study consisted of 4 consecutive 15-d periods made up of a 10-d diet adaptation, 4-d fecal collection, and 1 d for ruminal fluid sampling. As loads of feed were mixed daily for Exp. 3, the amount needed for Exp. 4 was removed from the beginning of each load; feed samples were analyzed independently from those in Exp. 3.

Steers were housed in individual outdoor pens (6.1 × 15.2 m). Each steer had ad libitum access to tank waterers, which were filled daily. Steers were fed once daily at approximately 1000 h. Diets were fed for ad libitum intake to target at least a 10% refusal. Total mixed diet samples were collected on days 10 through 14 and composited for each period for analysis. Overlapping individual ingredient samples from Exp. 3 coinciding with the sampling week were sent to SDK Laboratories for nutrient analysis (DM, ash, crude protein, ADF, NDF, starch, calcium, and phosphorus; Table 2). On days 4 through 14, Cr_2_O_3_ (10 g) was top-dressed and hand mixed into each animal’s diet as a marker to calculate digestibility. Refusals and fecal samples were collected on days 11 through 14, and ruminal fluid samples were collected on day 15. Refusal, fecal, and ruminal fluid samples were collected and analyzed following the same procedures as Exp. 2.

### Statistical Analysis

In Exp. 1, performance measures were analyzed with pen as the experimental unit using the MIXED procedure in SAS with the fixed effects of variety, processing, and variety × processing. Fecal starch parameters and net energy calculations were analyzed using the GLIMMIX procedure in SAS (v. 9.4), with the fixed effects of variety, processing, and variety × processing.

In Exp. 2, concentrations and proportions of VFA, ammonia, pH, and digestibility were analyzed in a linear mixed model fit in the GLIMMIX procedure with fixed effects of variety, processing, sampling hour, as well as their two- and three-way interactions. Period was included as a fixed effect, animal as a random effect, and sampling hour was modeled as a repeated measure with period × animal as the subject. The covariance structure for the repeated measures was selected from first order ante-dependent, compound symmetry, heterogeneous compound symmetry, unstructured, Toeplitz, and heterogeneous Toeplitz based on AIC values for each response variable.

In Exp. 3, performance measures and net energy calculations were analyzed with pen as the experimental unit using the MIXED procedure in SAS with the fixed effects of grain source, silage hybrid, and grain source × silage hybrid. Fecal starch parameters were analyzed using the GLIMMIX procedure in SAS with the fixed effects of grain source, silage hybrid, and grain source × silage hybrid. Silage DM and total starch were analyzed using the GLIMMIX procedure in SAS with the fixed effect of silage hybrid and date of sampling as a block.

In Exp. 4, ruminal parameters and digestibility were analyzed in a linear mixed model fit in the GLIMMIX procedure with fixed effects of grain source, silage hybrid, and sampling hour, as well as their two- and three-way interactions. Period was included as a fixed effect, animal as a random effect, and sampling hour was modeled as a repeated measure with period × animal as the subject. The covariance structure for the repeated measures was selected from first order ante-dependent compound symmetry, heterogeneous compound symmetry, unstructured, Toeplitz, and heterogeneous Toeplitz based on AIC values for each response variable.

Significance was considered as *P* < 0.05, trends as 0.05 < *P* < 0.10, and numerical differences as *P* > 0.10.

## Results and Discussion

### Experiment 1. Performance Study

Little morbidity and no mortality were observed in this experiment. One animal was treated for foot rot (CON/WS), one for bloat (EFC/WS), and one for pinkeye (EFC/DR); all animals recovered. Two steers were treated for chronic respiratory illness (CON/WS; EFC/WS) and removed from the research population as was one steer treated for lameness (CON/DR).

Performance results from Exp. 1 are presented in Table 4. There were no noteworthy treatment interactions observed for DM intake (DMI), average daily gain (ADG), or body weight. DMI of calves fed EFC tended to be lower (*P* < 0.09) than for calves fed CON over the entire 91-d trial. This difference was especially apparent through day 14, where CON-fed calves consumed more feed than their EFC-fed counterparts (*P* < 0.01). Over the entire 91-d trial, ADG and off-test weights tended to be greater (*P *< 0.10) for calves fed EFC, and G:F was 5.5% greater in calves fed EFC (*P* < 0.01). As early as day 35, G:F tended to be better for EFC-fed steers than for CON-fed steers (*P* < 0.07). For the remainder of the study (days 63 through 91), G:F was better for calves fed EFC (*P* < 0.02). These data agree with results published by Jolly-Breithaupt et al. (2019), which showed increases of 5.5% and 5.7% in G:F when feeding EFC containing an alpha-amylase enzyme as dry-rolled corn. However, another experiment by Jolly-Breithaupt (2018) revealed no differences in performance when cattle were fed dry-rolled EFC, which they suggested could be a result of specific growing conditions of the corn hybrid. Other researchers found no differences in DMI, ADG, or G:F when feeding a ground corn hybrid (CA3272) containing an alpha-amylase enzyme when included at 0%, 10%, or 20% of the diet DM (Schoonmaker et al., 2014). These researchers hypothesized that no differences were observed because the control diet allowed adequate capacity to hydrolyze starch due to the extensive level of corn processing and that the alpha-amylase enzyme may show better results in whole corn.

**Table 4.** Effect of Enogen Feed Corn and corn processing on performance and fecal composition (Exp. 1)

Item	Corn grain source^1^				SEM	P-value		
	CON		EFC					
	Corn processing2							
	DR	WS	DR	WS		Process	Source	Process × source
No. of pens	8	8	8	8				
No. of steers	95	95	96	93				
Body weight, kg								
Day 0	244	245	244	245	1.08	0.33	0.77	0.59
Day 7	258	259	259	259	1.91	0.70	0.80	0.85
Day 14	277	274	275	275	1.96	0.14	0.49	0.14
Day 35	307	305	307	306	2.53	0.17	0.32	0.54
Day 63	344	341	348	344	3.99	0.09	0.07	0.64
Day 77	360	360	367	364	6.89	0.62	0.10	0.68
Day 91	385	380	386	386	4.29	0.24	0.10	0.34
Daily gain, kg/d								
Days 0–7	2.08	2.04	2.07	2.07	0.28	0.87	0.93	0.91
Days 0–14	2.37	2.12	2.20	2.18	0.15	0.06	0.41	0.09
Days 0–35	1.80	1.71	1.80	1.77	0.07	0.07	0.37	0.38
Days 0–63	1.58	1.53	1.64	1.58	0.06	0.05	0.09	0.75
Days 0–77	1.51	1.49	1.59	1.55	0.09	0.52	0.11	0.75
Days 0–91	1.55	1.49	1.56	1.55	0.04	0.14	0.09	0.25
Dry matter intake, kg/d								
Days 0–7	6.62	6.57	6.45	6.24	0.25	0.31	0.03	0.55
Days 0–14	7.71	7.61	7.47	7.30	0.19	0.13	0.01	0.72
Days 0–35	8.58	8.54	8.56	8.12	0.30	0.09	0.11	0.14
Days 0–63	9.08	9.20	9.02	8.78	0.35	0.72	0.15	0.27
Days 0–77	9.13	9.34	9.03	8.90	0.37	0.83	0.11	0.32
Days 0–91	9.44	9.69	9.30	9.24	0.37	0.57	0.09	0.37
Gain:feed, kg/kg								
Days 0–7	0.314	0.311	0.325	0.332	0.020	0.92	0.44	0.81
Days 0–14	0.307	0.279	0.294	0.299	0.009	0.19	0.68	0.07
Days 0–35	0.210	0.201	0.211	0.218	0.005	0.81	0.07	0.10
Days 0–63	0.175	0.167	0.182	0.180	0.003	0.15	0.01	0.51
Days 0–77	0.165	0.160	0.176	0.174	0.005	0.48	0.02	0.76
Days 0–91	0.164	0.154	0.168	0.168	0.003	0.13	0.01	0.11
Diet NEm^3^, Mcal/kg	1.51	1.45	1.54	1.54	0.02	0.19	0.01	0.13
Diet NEg^4^, Mcal/kg	0.91	0.86	0.94	0.94	0.02	0.19	0.01	0.13
Fecal composition^5^								
Dry matter, %	18.8	19.9	16.7	17.9	0.39	0.01	<0.01	0.90
Starch, % of dry matter	12.0	21.8	6.1	13.8	1.41	<0.01	<0.01	0.45

CON, control (yellow #2 corn); EFC, Enogen Feed Corn.

DR, dry rolled; WS, whole shelled.

Net energy for maintenance, calculated as described by Galyean (2019) based on NRC (1996) requirements.

Net energy for gain, calculated as described by Galyean (2019) based on NRC (1996) requirements.

Fecal composition was measured on samples collected on day 56 or 57 of the 91-d experiment.

There were no effects of corn processing observed for overall DMI (*P* = 0.57) or ending body weight (*P* = 0.24). However, overall ADG (*P* < 0.14) and G:F (*P* < 0.13) were numerically better for DR than for WS. This supports research by Siverson et al. (2014) who fed similar diets and found no significant differences in performance between DR- and WS-corn when included at 29% of diet DM in diets containing 30% wet corn gluten feed.

There was a numeric (*P* = 0.11) source × processing interaction for overall G:F because the effect of processing (DR vs. WS) was greater for cattle fed CON than for those fed EFC. Cattle fed DR corn as EFC had 2.7% better G:F than those fed DR corn as CON. This is in general agreement with Jolly-Breithaupt (2018) where EFC yielded 2.2% greater G:F than CON when fed as DR corn. For our diets containing WS corn, the cattle receiving EFC had G:F 9.1% better than those fed CON. These results suggest that feeding EFC/WS resulted in performance of growing calves similar to that of calves fed DR of either variety. Thus, mastication by the animal may be sufficient to break down WS corn kernels containing the alpha-amylase trait, and processing of EFC may not be necessary to optimize digestion. Research by Beauchemin et al. (1994) supports this inference after observing that the majority of corn kernels were broken during the consumption and mastication of whole corn grain when it was fed to cows. When considering health, cost, and performance of the animal, the least amount of processing is best (Orskov, 1986).

The fecal starch analyses from days 56/57 (Table 4) show a processing effect with WS leading to a greater fecal starch concentration than DR (*P* < 0.01), suggesting less starch was digested and utilized by the animal when WS was fed. The EFC treatments led to lower starch concentrations in the feces than did CON (*P* < 0.01), suggesting a better starch digestibility for EFC. Fecal starch is a good indicator of starch digestion in cattle (Fredin et al., 2014; Zinn et al., 2007); although fecal starch concentrations can be affected by factors besides starch digestion, starch digestion would be expected to be the primary factor influencing fecal starch concentration for the diets used in Exp. 1 and 2.

Net energy values (Table 4) demonstrated significant differences between corn varieties. Diets containing the EFC treatments had greater concentrations of net energy for maintenance (NEm) and net energy for gain (NEg) than diets containing CON (*P* < 0.01). Net energy concentrations also demonstrated a numerical (*P* < 0.13) interaction between treatments because they demonstrated a pattern similar to that observed for G:F; NE values were greater for DR than for WS when CON was fed, but they were similar between DR and WS when EFC was fed. The dietary NE concentrations were less than what was originally formulated in the diets, which could reflect a number of factors that might have affected animal performance independent of the true dietary energy density.

In conclusion, G:F of calves receiving EFC was improved by 5.5% compared with calves receiving CON corn. This response became apparent by day 35 and was significant throughout the remainder of the study. There were no negative observations regarding the health or behavior of the calves when feeding EFC. By using a variety of corn that is more energy dense and requires less processing, producers can potentially increase economic efficiency of beef production.

### Experiment 2. Intake and Digestibility Study

Digestion and ruminal fermentation results are presented in Table 5. There were no effects of corn processing on digestion and ruminal parameters, no treatment interactions for digestibilities, and only a few tendencies for treatment interactions for ruminal parameters. The lack of effect for corn processing generally agrees with Exp. 1 and reiterates the results of Siverson et al. (2014) and Beauchemin et al. (1994) where no differences in digestibility were found between DR and WS corn.

**Table 5.** Effects of Enogen Feed Corn and processing on total tract digestibility and ruminal characteristics (Exp. 2)

Item	Corn grain source^1^				SEM^3^	*P*-value		
	CON		EFC					
	Corn processing^2^							
	DR	WS	DR	WS		Process	Source	Process × source
No. of observations	7	7	6	7				
Dry matter intake, kg/d	8.21	7.68	7.75	8.14	0.43	0.80	0.99	0.11
Ruminal								
pH^4^	5.81	5.93	5.84	5.87	0.06	0.15	0.82	0.37
Ammonia, mM^4^	2.79	2.38	3.63	2.80	0.73	0.32	0.30	0.73
Total VFA, mM^4^	109.4	107.0	102.1	109.5	5.27	0.45	0.45	0.14
Acetate, molar %^4^	62.0	61.4	60.8	60.3	1.33	0.68	0.34	0.95
Propionate, molar %^4^	24.5	25.1	24.4	26.4	1.38	0.33	0.65	0.60
Butyrate, molar %^4^	9.7	9.5	10.6	9.5	0.60	0.24	0.41	0.36
Isobutyrate, molar %^4^	1.22	1.38	1.40	1.30	0.07	0.62	0.47	0.07
Valerate, molar %^4^	1.42	1.45	1.69	1.44	0.09	0.18	0.10	0.09
Isovalerate, molar %^4^	1.11	1.13	1.17	1.06	0.12	0.65	0.99	0.52
Liquid passage rate, %/h	9.5	8.8	7.4	8.4	0.67	0.77	0.01	0.09
Digestibility, %								
Dry matter	58.4	56.2	62.0	63.2	2.53	0.83	0.04	0.50
Organic matter	61.5	59.3	64.8	66.0	2.45	0.82	0.04	0.46
Neutral detergent fiber	51.1	47.0	51.5	53.5	4.18	0.79	0.41	0.46
Acid detergent fiber	48.5	42.0	50.0	54.5	5.20	0.85	0.17	0.28
Starch	84.7	85.1	86.4	90.4	2.90	0.37	0.16	0.47

CON, control (yellow # corn corn); EFC, Enogen Feed Corn.

DR, dry rolled; WS, whole shelled.

Largest value among treatments reported.

Average of values collected at 0, 2, 4, 6, 8, 12, 18, and 24 h after feeding on day 15 of each period.

**Figure 1.**
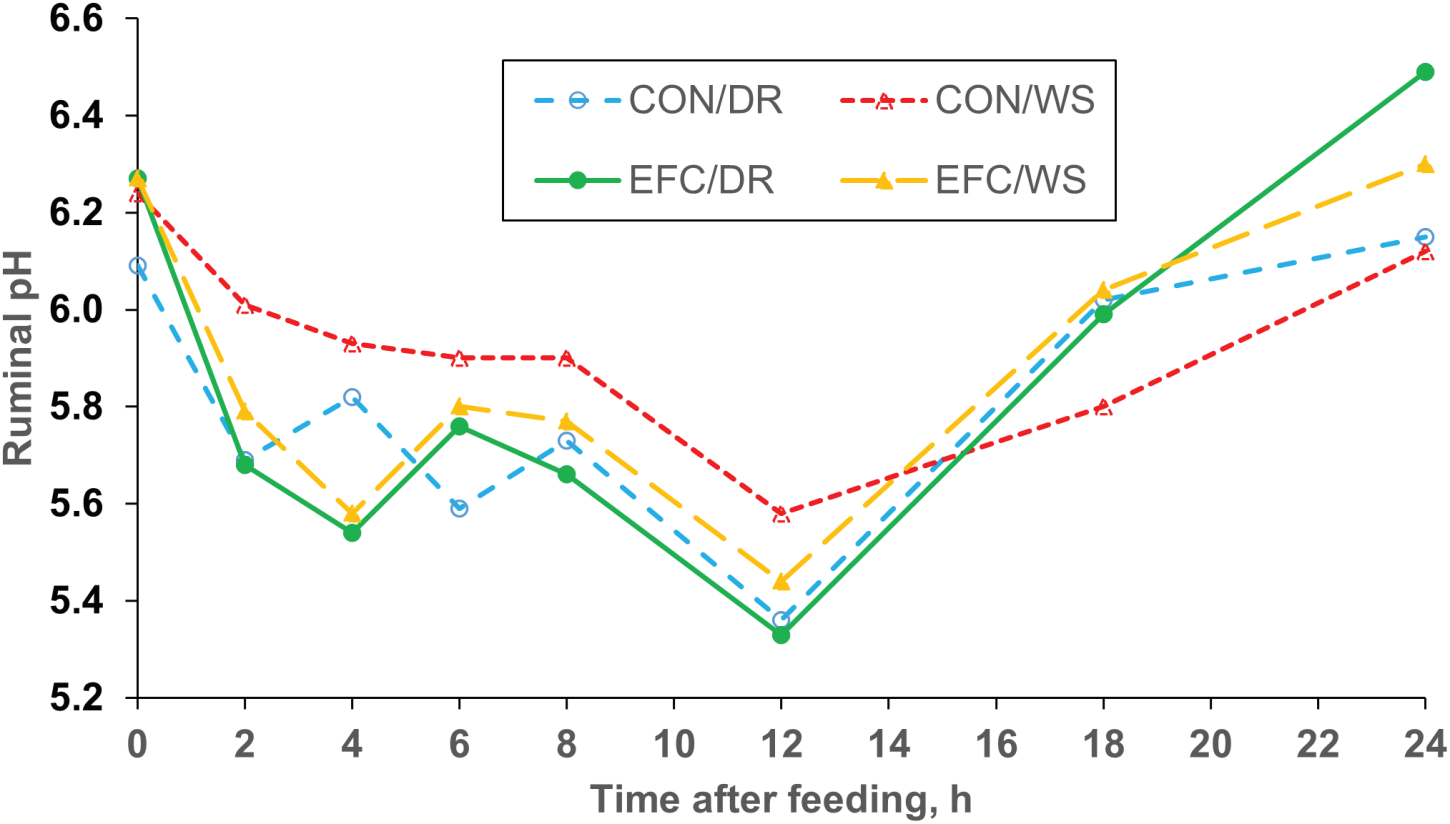
Effects of Enogen Feed Corn grain on ruminal pH measured over 24 h (Exp. 2). CON/DR = Yellow #2 corn grain/dry-rolled. CON/WS = Yellow #2 corn grain/whole-shelled. EFC/DR = Enogen Feed Corn grain/dry-rolled. EFC/WS = Enogen Feed Corn grain/whole-shelled. Corn (*P* = 0.82), processing (*P* < 0.15), corn × processing (*P* < 0.37), hour (*P* < 0.0001), hour × corn (*P* < 0.01), hour × processing (*P* = 0.40), hour × corn × processing (*P* = 0.64).

Averaged over time, ruminal pH (*P* > 0.82) and ruminal ammonia concentrations (*P* > 0.30) were not affected by corn variety (Table 5). However, ruminal pH demonstrated a corn source × time after feeding interaction (Figure 1). This interaction demonstrated that decreases in ruminal pH between pre-feeding values and nadir values obtained at 12 h after feeding were greater for steers fed EFC than for those fed CON, suggesting a more rapid fermentation of EFC than of CON. It is also possible that differences in feed consumption patterns contributed to this effect.

Liquid passage rate was faster for CON-fed calves than for EFC-fed calves (*P* < 0.01), which might in part explain the tendency for DMI to be greater for calves fed CON than for EFC in Exp. 1. However, DMI was not affected by treatment in Exp. 2. Passage rate can be inversely related to digestibility because faster passage allows less time for ruminal digestion, and the faster liquid passage rate for calves fed CON than for those fed EFC was associated with lower digestibilities for CON than for EFC. The relationships between liquid passage rate and digestibility do not provide evidence of causation, although one possible linkage is that faster fermentation rates for EFC than for CON created periods post-feeding where ruminal pH was lower, and this may have reduced ruminal contractions and ultimately slowed ruminal passage (González et al., 2012).

Total tract DM and OM digestibilities were greater for EFC-fed calves than for CON-fed calves (*P* < 0.04), representing 8% and 9% increases, respectively. More energy should be available to the animal when digestibility increases, so the differences in digestibility between EFC and CON may explain differences in G:F observed in Exp. 1. In agreement with this study, Jolly-Breithaupt (2018) observed tendencies for increases in total tract digestibility of DM and OM with the feeding of EFC, and they also observed significantly greater (4.2%) total tract starch digestibility with EFC; in our study, the greater starch digestion for EFC than for CON was not statistically significant but was of the same magnitude observed by Jolly-Breithaupt (2018) and large enough (4.2%) to be biologically important. Rojo et al. (2005) supplemented alpha-amylase from *Bacillus licheniformis* to lambs, observing an increase in total tract DM, OM, and starch digestion.

The digestibility trial was designed to determine if the effects of treatments on growth efficiency in Exp. 1 could be explained by differences in total tract digestibility. Treatment effects on performance in Exp. 1 and digestion measures in Exp. 2 showed some similarities, such as greater performance and digestion for heifers fed EFC rather than CON, but the numerical interaction between treatments for G:F in Exp. 1 was not mimicked by digestibilities in Exp. 2.

Volatile fatty acid concentrations are shown in Table 5. Total VFA concentrations were not affected by treatment. Previous research involving supplementing exogenous alpha-amylase in cattle diets has been extremely variable. Researchers have either discovered an increase in acetate (Tricarico et al., 2005; Rojo et al., 2005), an increase in propionate (Nozière et al., 2014), or found no effects on ruminal VFA concentrations in ruminant animals supplemented with alpha-amylase (Jolly-Breithaupt, 2018; Hristov et al., 2008). Molar percentages of acetate, propionate, butyrate, and isovalerate were not affected by treatment (*P* > 0.24). The molar percentage of valerate tended to be greater for EFC treatments (*P *< 0.10), and this resulted from EFC/DR having greater valerate percentages than the other three treatments (processing × source interaction; *P* < 0.09). Isobutyrate percentage tended to be greatest for the EFC/DR treatment as well (*P* < 0.07). When analyzing in vitro fermentation of steam-flaked corn, Horton et al. (2020) observed increases in valerate production as the percentage of EFC grain increased in corn grain mixes.

### Experiment 3. Performance Study

Little morbidity and no mortality were observed for this experiment. One animal was treated for respiratory illness (EFC-G/EFC-S) and two were treated for bloat (CON-G/EFC-S; CON-G/CON-S); all animals recovered. Two steers were also treated for lameness and removed from the experiment (CON-G/CON-S; EFC-G/EFC-S).

Performance results from Exp. 3 are shown in Table 6. No significant effects of corn grain source were noted for the overall 91-d feeding trial, nor were any significant interactions between corn silage hybrid and corn grain source observed.

**Table 6.** Effects of Enogen Feed Corn silage and corn on performance (Exp. 3)

Item	Corn silage hybrid^1^				SEM	*P*-value		
	CON-S		EFC-S					
	Corn grain source^2^							
	CON-G	EFC-G	CON-G	EFC-G		Grain	Silage	Grain × Silage
No. of pens	8	8	8	8	—	—	—	—
No. of animals	88	87	87	88	—	—	—	—
Body weight, kg								
Day 0	301	299	297	297	—	—	—	—
Day 14	334	336	335	336	3.37	0.25	0.71	0.97
Day 28	345	343	343	341	5.51	0.47	0.47	0.80
Day 42	375	372	374	374	4.10	0.61	0.93	0.38
Day 56	395	393	395	396	4.37	0.81	0.62	0.35
Day 70	415	413	415	416	5.73	0.90	0.50	0.62
Day 77	420	423	426	426	5.84	0.66	0.09	0.56
Day 91	429	427	433	433	5.94	0.77	0.10	0.85
ADG, kg/d								
Days 0–14	2.35	2.63	2.69	2.80	0.21	0.06	0.02	0.39
Days 0–28	1.55	1.58	1.66	1.56	0.19	0.70	0.67	0.47
Days 0–42	1.76	1.75	1.83	1.83	0.09	0.96	0.07	0.76
Days 0–56	1.68	1.68	1.74	1.76	0.08	0.84	0.06	0.70
Days 0–70	1.62	1.63	1.68	1.69	0.08	0.83	0.08	0.95
Days 0–91	1.40	1.41	1.49	1.48	0.07	0.97	0.01	0.82
Dry matter intake, kg/d								
Days 0–14	7.68	8.01	7.69	7.90	0.13	0.05	0.69	0.66
Days 0–28	8.13	8.40	8.19	8.42	0.14	0.09	0.76	0.92
Days 0–42	8.48	8.77	8.81	8.97	0.14	0.12	0.07	0.63
Days 0–56	8.86	9.05	9.18	9.35	0.14	0.21	0.03	0.95
Days 0–70	9.14	9.23	9.38	9.52	0.14	0.42	0.08	0.84
Days 0–77	9.20	9.43	9.48	9.64	0.14	0.18	0.09	0.78
Days 0–91	9.17	9.38	9.44	9.56	0.12	0.19	0.08	0.71
Gain:feed, kg/kg								
Days 0–14	0.306	0.328	0.350	0.354	0.011	0.24	<0.01	0.45
Days 0–28	0.192	0.189	0.203	0.185	0.010	0.33	0.74	0.48
Days 0–42	0.192	0.184	0.181	0.182	0.004	0.19	0.64	0.45
Days 0–56	0.191	0.186	0.190	0.189	0.004	0.48	0.84	0.68
Days 0–70	0.178	0.176	0.180	0.178	0.004	0.67	0.60	0.96
Days 0–77	0.168	0.171	0.177	0.174	0.004	0.88	0.16	0.47
Days 0–91	0.155	0.152	0.160	0.157	0.002	0.43	0.14	0.94
NEm^3^, Mcal/kg	1.61	1.58	1.62	1.60	0.02	0.31	0.42	0.89
NEg^4^, Mcal/kg	1.00	0.97	1.01	0.99	0.01	0.30	0.39	0.87
Fecal composition^5^								
Dry matter,%	18.6	19.3	18.6	19.2	0.68	0.38	0.99	0.94
Starch, % of dry matter	20.4	21.7	19.6	23.5	2.00	0.20	0.82	0.52

CON-S, control corn silage (Mycogen); EFC-S, Enogen Feed Corn silage.

CON-G, control corn grain (yellow #2 corn); EFC-G, Enogen Feed Corn grain.

Net energy for maintenance, calculated as described by Galyean (2019) based on NRC (1996) requirements.

Net energy for gain, calculated as described by Galyean (2019) based on NRC (1996) requirements.

Fecal composition was measured on samples collected on day 49 of the 91-d experiment.

Over the entire trial (days 0 to 91), ADG was greater for EFC-S (*P* < 0.01) than for CON-S, and this effect demonstrated significance or tendencies throughout most of the trial’s time points. There was a corresponding tendency for ending body weight to be greater for EFC-S than for CON-S (*P* = 0.10).

DMI tended to be greater (*P* < 0.08) for calves fed EFC-S over the entire 91-d trial, and this tendency became apparent as early as day 42 of the trial. Lara et al. (2018b) researched effects of feeding corn silage diets with or without an amylolytic enzyme supplemented to lambs. They found that providing 602 dextrinizing units of alpha-amylase/kg DM in the total mixed ration had no effect on DMI or ADG, and, although not significant, G:F was improved by 4.8% for lambs fed corn silage with an alpha-amylase supplement. Over the first 28 d of the experiment, DMI was greater (*P* ≤ 0.09) for steers fed EFC-G compared with those fed CON-G, but this effect lost its significance as the trial progressed.

G:F over the full 91-d study was numerically better in calves fed EFC-S than in those fed CON-S (*P* = 0.14), with improvements of 3.3%. This effect was not apparent until day 77 of the trial. In agreement with the performance results in our experiment, Leahy et al. (1990) observed an increase in ADG (*P* < 0.01), G:F (*P* < 0.01), and ending body weight (*P* < 0.05) in beef heifers when fed corn silage treated with alpha-amylase at 0.05% (wet basis) before ensiling, resulting in 11% increases in performance in ADG and G:F for heifers fed the alpha-amylase treatment.

Results from the day 49 fecal sampling showed no effects of corn grain source or silage hybrid on fecal starch concentration (Table 6). On the surface, this might suggest that there were no differences in starch digestion. However, at feeding, starch concentration of the EFC silage was greater than that of the CON silage (34.7% vs. 28.7%; *P* < 0.01), so differences in starch intake may also have influenced fecal starch concentrations. Additionally, the EFC silage had a greater DM concentration than the CON silage (34.4% vs. 30.0%; *P *< 0.01). The differences in silage composition may have played a role in the performance differences between silages. In trials such as ours, it is difficult to fully evaluate the effects of forage variety because agronomic, processing, and storage factors play a role in the nutrient profile of the final forage.

Dietary net energy concentrations calculated from growth performance did not differ among treatments (Table 6). Given the expectation of a strong relationship between G:F and dietary net energy density, particularly in a study such as ours where initial body weights were tightly controlled and variation in feed intake among treatments was relatively minor, the inability of our experiment to detect differences among treatments for net energy was surprising. However, treatment effects on G:F were not highly significant, and nuanced differences between G:F and net energy for treatment responses can explain the lack of treatments effects for net energy. The net energy concentrations calculated from performance were less than formulated for the diets, which could relate to the many factors beyond energy intake that can influence cattle performance.

Overall, G:F of calves receiving EFC-S was improved by 3.3% and ADG improved by 6.0% compared with calves receiving CON-S. No significant effects of corn grain source were noted over the entire 91-d trial, nor were any overall significant interactions between corn silage type and corn grain type. There were no negative observations regarding cattle health or behavior with the feeding of EFC silage.

### Experiment 4. Intake and Digestibility Study

Effects of corn grain source and corn silage hybrid on ruminal characteristics and total tract digestion are presented in Table 7. With the exception of ruminal proportions of several VFA, we observed no interactions between corn grain source and corn silage hybrid.

**Table 7.** Effects of Enogen Feed Corn grain and silage on total tract digestibility and ruminal characteristics (Exp. 4)

Item	Corn silage hybrid^1^				SEM^3^	*P*-value		
	CON-S		EFC-S					
	Corn grain source^2^							
	CON-G	EFC-G	CON-G	EFC-G		Grain	Silage	Grain × silage
Number of observations	7	8	8	8				
Dry matter intake, kg/d	7.91	7.93	7.46	7.18	0.51	0.49	<0.01	0.41
Ruminal								
pH^4^	6.37	6.47	6.32	6.37	0.09	0.27	0.23	0.67
Ammonia, mM^4^	3.92	3.45	3.87	2.94	0.45	0.06	0.44	0.51
Total VFA, mM^4^	106.9	106.6	113.4	111.3	5.52	0.82	0.27	0.85
Acetate, molar %^4^	64.9	64.6	60.8	64.3	0.77	0.03	<0.01	0.01
Propionate, molar %^4^	19.2	19.9	23.8	19.9	0.78	0.03	<0.01	<0.01
Butyrate, molar %^4^	10.9	10.9	11.0	10.9	0.61	0.88	0.81	0.76
Isobutyrate, molar %^4^	1.21	1.22	1.24	1.25	0.09	0.90	0.73	0.96
Valerate, molar %^4^	1.32	1.20	1.24	1.23	0.06	0.17	0.54	0.20
Isovalerate, molar %^4^	2.50	2.14	1.87	2.28	0.16	0.78	0.11	0.01
Liquid passage rate, %/h	13.1	14.2	13.5	13.4	0.66	0.32	0.62	0.20
Digestibility, %								
Dry matter	65.3	64.7	67.0	66.3	1.77	0.64	0.24	0.98
Organic matter	67.7	67.1	69.2	68.6	1.73	0.66	0.26	0.97
Neutral detergent fiber	58.6	60.2	61.0	60.6	2.01	0.75	0.44	0.56
Acid detergent fiber	59.7	60.7	61.6	60.5	2.04	0.96	0.63	0.58
Starch	84.6	83.5	85.8	83.9	2.51	0.31	0.57	0.76

CON-S, control corn silage (Mycogen); EFC-S, Enogen Feed Corn silage.

CON-G, control corn grain (yellow #2 corn); EFC-G, Enogen Feed Corn grain.

Largest value among treatments reported.

Average of values collected at 0, 2, 4, 6, 8, 12, 18, and 24 h after feeding on day 15 of each period.

Feeding CON-S led to greater DMI than feeding EFC-S (*P* < 0.01). This is notably opposite of the effect observed in the corresponding performance study (Exp. 3).

**Figure 2.**
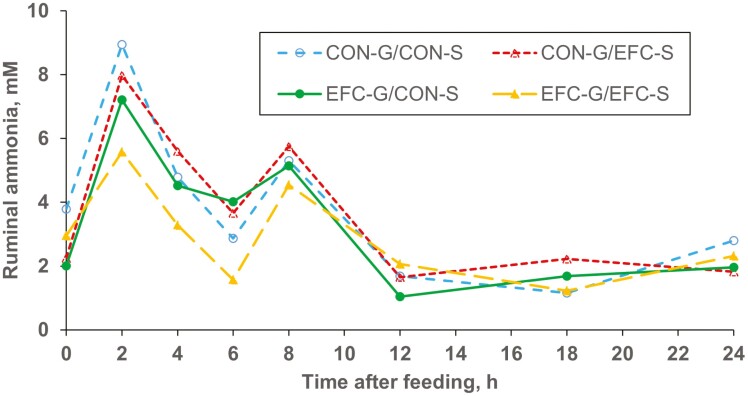
Effects of Enogen Feed Corn silage and grain on ruminal ammonia measured over 24 h (Exp. 4). CON-G = Yellow #2 corn grain; CON-S = Mycogen corn silage. EFC-G = Enogen Feed Corn grain; EFC-S = Enogen Feed Corn silage. Corn grain (*P* < 0.06), corn silage (*P* = 0.44), corn grain × corn silage (*P* = 0.51), hour (*P* < 0.0001), hour × corn grain (*P* = 0.91), hour × corn silage (*P* = 0.87), hour × corn grain × corn silage (*P* < 0.01).

There were no effects of corn grain source, silage hybrid, or grain source × silage hybrid interactions for liquid passage rate (*P* > 0.20), ruminal pH (*P* > 0.23), or digestibilities of DM, OM, NDF, ADF, or starch (*P* > 0.24). CON-G tended to lead to higher ammonia concentrations than EFC-G (*P* < 0.06), which could be attributable either to greater degradation of dietary protein provided by CON-G than by EFC-G or to greater microbial capture of ammonia when the EFC-G was fed. Although total tract starch digestion was not affected by treatment, it is possible that ruminal starch digestion was greater for EFC-G than for CON-G, and the lower ruminal ammonia concentrations for EFC-G would support this possibility. Ruminal ammonia concentrations measured over time post-feeding are presented in Figure 2. There was a grain source × silage hybrid × time interaction, because ruminal ammonia did not increase as much after feeding for EFC-G/EFC-S as for the other three treatments. As noted above, this could relate to either ruminal degradation of dietary protein or microbial capture of ammonia.

Ruminal concentrations of total VFA (Table 7) were not affected by grain source, silage hybrid, or an interaction between them (*P* > 0.35). Interactions between grain source and silage hybrid were observed for molar percentages of propionate and acetate, because steers fed CON-G/EFC-S had greater proportions of propionate but lesser proportions of acetate than steers fed the other three treatments (grain × silage interactions; *P* ≤ 0.01). Molar proportions of butyrate, isobutyrate, and valerate, when averaged over all sampling times, were not affected by treatment. Calves fed the CON-G/CON-S treatment had the highest molar percentages of isovalerate, whereas calves fed CON-G/EFC-S had the lowest proportions (*P* < 0.01). Both valerate and isovalerate demonstrated grain source × silage hybrid × time interactions (*P* ≤ 0.04) because their concentrations increased more over the initial 8 h after feeding for CON-G/CON-S and EFC-G/EFC-S than for EFC-G/CON-S or CON-G/EFC-S (data not shown).

Lara et al. (2018a) evaluated effects of feeding corn silage diets with or without an amylolytic enzyme supply to cannulated wethers; when providing 602 dextrinizing units of alpha-amylase/kg DM in the total mixed ration, molar proportions of propionic acid increased. Horton (2018) studied ensiled high-moisture EFC grain using in vitro ruminal fermentations. They observed greater production of butyrate and total VFA for high-moisture EFC than for high-moisture control corn (*P* < 0.05). Both Lara et al. (2018a) and Horton et al. (2020) clearly demonstrate that added amylase and amylase provided by corn itself can influence ruminal VFA profiles. Our data similarly showed that ruminal VFA profiles can be affected by feeding EFC, but our results did not show a consistent effect of amylase provided through grain or silage, and effects were not additive when the amylase was provided from both grain and silage simultaneously. Further investigation into effects on VFA profiles are warranted.

Total tract digestibilities did not demonstrate any significant treatment responses (*P* > 0.24), but numerical differences showed greater total tract digestibilities of DM (2.5%) and OM (2.2%) for EFC-S than for CON-S; this could explain the increased performance of calves fed EFC-S in Exp. 3. It is possible that the greater starch content of the EFC-S relative to CON-S may have been more important in creating this response than the presence of alpha-amylase, but it is impossible to directly separate these factors. Jolly-Breithaupt (2018) compared feeding dry-rolled corn from either EFC-G or as CON-G with either wet corn gluten feed or modified distillers grains in diets containing 15% control corn silage; feeding EFC-G led to greater digestion of DM, OM, and starch than did the feeding of CON-G, and this response was consistent for diets containing wet corn gluten feed or modified distillers grains. Lara et al. (2018a) observed an increase in apparent OM and DM digestibility by wethers when corn silage was supplemented with an alpha-amylase enzyme in the diet. In Exp. 2, we observed improvements in digestion of DM and OM when EFC-G was supplemented, whereas in Exp. 4 the feeding of EFC-G did not lead to even numerical increase in DM or OM digestion. Taken as a whole, increases in amylase activity appear to increase digestion of DM, OM, and starch, but Exp. 4 did not demonstrate these responses when the amylase was provided from EFC-G and only numerical effects on digestion were observed when amylase was provided from EFC-S. These observations on total tract digestion generally follow the effects observed in the companion performance trial (Exp. 3) where EFC-S improved performance but EFC-G did not.

### Implications

There were no negative observations regarding the health or behavior of the calves when feeding EFC-G or EFC-S. Relative to control corn, there were significant advantages in G:F when feeding EFC grain in Exp. 1 and EFC-S in Exp. 3. In contrast, EFC-G did not improve performance or digestion in Exp. 3 and 4. Under our experimental circumstances, cattle fed whole-shelled EFC grain had performance that was as good as those fed dry-rolled EFC or dry-rolled CON corn grain, whereas cattle fed whole-shelled CON corn grain had worse performance. Thus, feeding EFC as whole-shelled grain has the potential to be beneficial to the stocker/grower sector of the beef industry by eliminating processing costs without sacrificing performance or digestibility. Digestibility of the corn grain was increased with the addition of the alpha-amylase enzyme present in EFC. Overall, the results of these studies indicate that using a hybrid of corn containing an alpha-amylase enzyme generally improved G:F in growing calves.
